# Dr. Noe’s Case in June Number

**Published:** 1891-07

**Authors:** Wm. Steinrauf

**Affiliations:** St. Charles, Mo.


### DR. NOE'S CASE IN JUNE NUMBER.

If Dr. A. T. Noe will examine his lady patient closely, for whom the advice is wanted in the last number of THE HOMŒOPATHIC PHYSICIAN, he will discover, I think, that there is ovarian trouble. Although we do not treat ovarian disease *per se*, still a physician should know where there is irritation and what organ is affected, if for no other reason than to satisfy the friends.

There are a good many symptoms surely omitted from the doctor's "Advice Wanted," still I think if he will study and consult such remedies as Apis, Cimicifuga, Lilium-tigr., Nux-vomica, etc., I believe he may find something to benefit Mrs. L. F——.

From his description of the case I should prescribe Cimicifuga.

WM. STEINRAUF, M. D.,

June 2d, 1891. St. Charles, Mo.

